# Advanced Artificial Muscle for Flexible Material‐Based Reconfigurable Soft Robots

**DOI:** 10.1002/advs.201901371

**Published:** 2019-09-04

**Authors:** Zhongdong Jiao, Chao Zhang, Wei Wang, Min Pan, Huayong Yang, Jun Zou

**Affiliations:** ^1^ State Key Laboratory of Fluid Power and Mechatronic Systems Zhejiang University Hangzhou 310027 China; ^2^ Centre for Power Transmission and Motion Control Department of Mechanical Engineering University of Bath Bath BA2 7AY UK; ^3^ Ningbo Research Institute Zhejiang University Hangzhou 315100 China

**Keywords:** artificial muscle, building bricks, flexible materials, origami‐inspired design, reconfigurability, soft robots

## Abstract

Flexible material‐based soft robots are widely used in various areas. In many situations, the suitable soft robots should be rapidly fabricated to complete the urgent tasks (such as rescue), so the facile fabricating methods of the multifunctional soft robots are still in urgent needs. In this work, the origami structure is employed to design vacuum‐powered silicone rubber artificial muscles, which can perform multiple motions, including contraction, bending, twisting, and radial motions. Artificial muscles can be used for rapid reconfiguration of different soft robots, just like the “building bricks”. Based on these artificial muscles, four soft robots with different functions, including an omnidirectional quadruped robot, a flexible gripper, a flexible wrist, and a pipe‐climbing robot, are reconfigured to complete different tasks. The proposed origami artificial muscles offer a facile and rapid fabricating method of flexible material‐based soft robots, and also greatly improve the utilization rate of flexible materials.

## Graphical abstract

**The origami structure** is employed to design vacuum‐powered silicone rubber artificial muscles, which can perform multiple motions. Artificial muscles can be used for rapid reconfiguration of different soft robots, just like the “building bricks”. The robots allow simpler, less expensive, modular units to be reconfigured into a group depending on the task that needs to be completed.

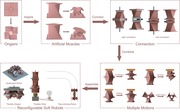

Flexible materials are widely used in soft robotics,^1^, ^2^, ^3^, ^4^, ^5^ flexible sensors,^6^, ^7^, ^8^, ^9^ and wearable devices^10^, ^11^ due to their low weight, high flexibility, and large deformation. Particularly, in soft robotics, artificial muscles made by flexible materials can perform contraction,^12^, ^13^ bending,^2^, ^13^, ^14^, ^15^ twisting,^13^, ^16^, ^17^ helical motions,^18^, ^19^ etc. Compared to motor‐based robots, flexible material‐based soft robots have great advantages in achieving various locomotions, manipulating delicate objects, providing safer human–robot interaction, and adapting to confined environments.

To date, scientists and engineers mainly focus on the development of new flexible materials or their certain properties (e.g., stretchability), thereby improving the abilities of flexible material‐based soft robots. Although soft robots become more powerful and stronger in some ways, little attention is paid to their reconfigurability. Generally, each soft robot has a unique shape and function, and new soft robots should be designed and fabricated to solve a current task. Metaphorically speaking, a pair of scissors is used to cut things and different from a hammer which is meant to hit. Even if they are made of the same material, neither can perform two tasks simultaneously. This leads to the redundancy of materials. Moreover, in many situations, such as exploration, searching, and rescue, knowledge of the task to be performed, or the context in which it is to be performed, cannot be known a priori. Thus, it is impossible to manufacture suitable soft robots in advance so as to complete the task smoothly. In such an emergency or lack of resources, the reconfigurability of soft robots is very important and meaningful. The reconfigurable soft robots can cope with different situations and save a lot of time. Is it possible to create reconfigurable soft robots that can reshape for different tasks?

Origami, the ancient art of paper folding, attracts great attention of scientists and engineers owing to its structural reconfigurability and multistability. On one hand, origami folding is an effective way to reconfigure the shape, on the other hand, bistability and multistability have been identified in certain origami patterns such that by switching among different stable states the structural mechanical properties can be reversibly reprogrammed. Due to these notable advantages, various origami‐inspired structures have been exploited in metamaterials,^20^, ^21^ self‐folding structures,^22^, ^23^ microelectromechanical systems,^24^, ^25^ and rigid robots.^26^, ^27^ Obtaining inspiration from origami may be an effective way for the better structure design of flexible material‐based artificial muscles in soft robotics, thereby realizing the “building bricks” of soft robots. Nevertheless, little work has been focused on this issue.

In this study, we were inspired by the origami structure, and thus used it to design vacuum‐powered silicone rubber artificial muscles for the reconfiguration of soft robots. Two novel origami artificial muscles with the same appearance and different inner structures were fabricated. The characteristics, motions, and performances of these artificial muscles were investigated. Then, several combinations of artificial muscles and their motions were discussed. Finally, various motions were demonstrated by four reconfigurable robots: a modular quadruped robot, a flexible gripper, a flexible wrist, and a pipe‐climbing robot.

*Characteristics*: The origami structure of artificial muscles is shown in **Figure** **1**, and two kinds of artificial muscles are presented: twisting‐contraction artificial muscle (TCAM) and twisting‐bending artificial muscle (TBAM). The structure of TCAM is shown in Figure 1a–d, where two square facets and four curved facets form a monolithic chamber, and a silicone tube is plugged into the chamber to change its interior pressure. The pre‐twisted angle α between the top and bottom facets leads to the pre‐twisted structure (Figure 1c). The mountain creases are replaced with slanted sides which are thicker silicone rubber (5 mm) than facets (2.5 mm), while the valley creases are positioned in the middle of two mountain creases and only visible when the artificial muscle is actuated (the dotted line in Figure 1b). In addition, the four holes, designed in the top and bottom facets, are used to connect with other artificial muscles or devices (Figure 1d). The TBAM has an identical appearance with TCAM except there is an asymmetric silicone rubber infill in the chamber (Figure 1e), where the filling angle γ between the top facet and the inclined bottom of the infill is used to describe the amount of the infill.

**Figure 1:**
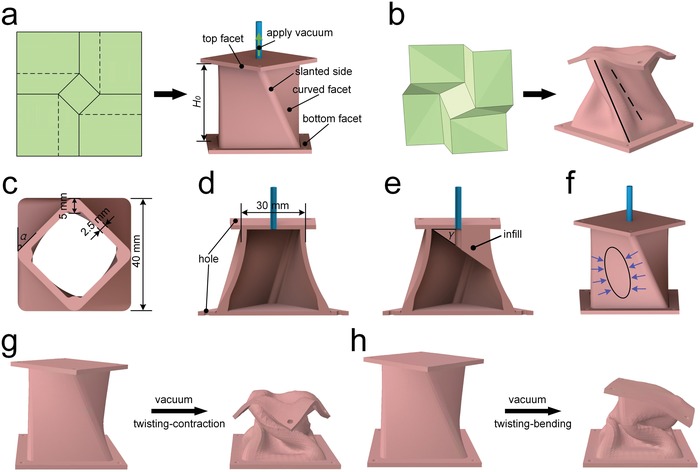
Origami‐inspired structure of TCAM and TBAM. a) The artificial muscle inspired from square‐twist origami. *H*_0_ is the height of artificial muscle. The solid lines indicate mountain creases and the dotted lines indicate valley creases. b) An actuated state of origami‐inspired artificial muscle. c) Top view of the TCAM. α is the pre‐twisted angle between the top and bottom facets. d) The inner structure of the TCAM. e) The inner structure of the TBAM. γ is the filling angle between the top facet and the inclined bottom of the infill. f) The tensions that curved facet is subjected to when the TCAM is evacuated. g) The clockwise TCAM is actuated by a vacuum. h) The clockwise TBAM is evacuated.

*Motions*: TCAM and TBAM have a compound motion: twisting‐contraction and twisting‐bending, respectively. As shown in Video S1 (Supporting Information), the thin‐walled chamber is deformed and the elastic energy is stored when the TCAM is evacuated. The uneven thickness and the pre‐twisted structure lead to the different levels of deformation in different parts of the chamber. The curved facets, the thinnest parts of the chamber, collapse inward and form concavities at their central position (the black ellipse in Figure 1f). Different from the TCAM, the TBAM with a chamber filled with some silicone rubber will show smaller deformation when evacuated. The asymmetric contraction of the four curved facets causes the artificial muscle to bend to the side with the least infill and twist to its maximum angle (Figure 1h).

*Performances*: The deformation performances of artificial muscles are tested. **Figure** **2**a shows the influence of actuating pressure on twisting angle and contraction of TCAM. It is apparent that the contraction and twisting angle increase with the increase of actuating pressure. The relationship between muscle deformations (contraction and twisting angle) and pressure is nonlinear, and no obvious hysteresis between the actuating and the releasing processes is observed. The influence of actuating pressure on twisting angle and bending angle of TBAM is nonlinear, as shown in Figure 2b. It is observed that the twisting angle always increases with the increase of actuating pressure, while the bending angle increases initially, then decreases with the increase of actuating pressure. This variation of bending angle is due to that the curved facets without silicone rubber infill collapse earlier than those with silicone rubber infill. Moreover, no obvious hysteresis between the actuating and the releasing processes can also be observed. The artificial muscles have good shape recovery and deformation performances in cycle experiments, as shown in Figure 2c,d and Tables S3 and S4 (Supporting Information). It takes 0.27 and 0.62 s to reach its deformed state and return to its original state for TCAM, while it takes 0.25 and 0.55 s to twist to its deformed state and return to its original state for TBAM. The deformation response of TBAM is faster than that of TCAM due to the presence of silicone rubber infill which decreases the chamber volume of TBAM. All these phenomena indicate that the origami artificial muscles have excellent deformation performances.

**Figure 2:**
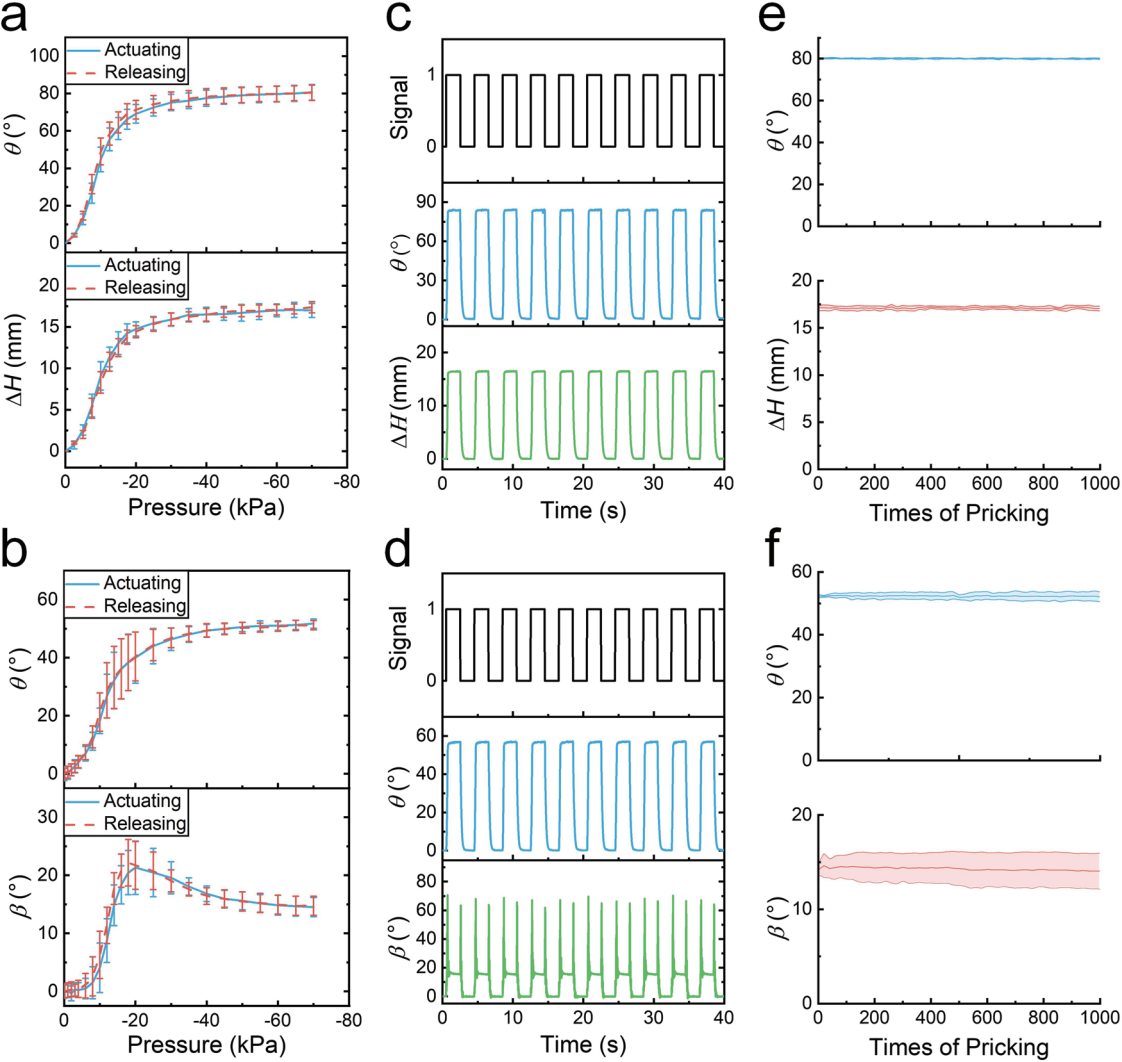
Performances of artificial muscles. a) The twisting angle θ and contraction ∆*H* of TCAM as a function of actuating pressure. b) The twisting angle θ and bending angle β of TBAM as a function of actuating pressure. Error bars indicate the SD for *n* = 9 measurements at each data point. c) The twisting angle θ and contraction ∆*H* dynamic responses of TCAM. The operation pressure (signal = 1) is −90 kPa and the releasing pressure (signal = 0) is 0 kPa. d) The twisting angle θ and bending angle β dynamic responses of TBAM. The operation pressure (signal = 1) is −90 kPa and the releasing pressure (signal = 0) is 0 kPa. e) Twisting angle θ and contraction ∆*H* of TCAM as a function of the times of pricking with a needle. f) Twisting angle θ and bending angle β of TBAM as a function of the times of pricking with a needle.

In many situations, the pointed objects like nail or sharp stone may be a threat to artificial muscles in soft robotic applications. Artificial muscles with good damage resistance are necessary. Herein, the damage experiments of artificial muscles are carried out by hammer hit and needle prick. As shown in Video S2 (Supporting Information), artificial muscles can still function normally after being hit by a hammer and pricked with a needle. The influence of the times of pricking on the artificial muscles is shown in Figure 2e,f, where the deformation of artificial muscles has slight change with the times of pricking increases. All these results demonstrate that the origami‐inspired artificial muscles have good damage resistance performances, which can improve the adaptability of soft robots to unknown environment.

*Connection Methods of Artificial Muscles*: Two connection methods, rigid connection and soft connection, are designed to rapidly connect artificial muscles so as to reconfigure various soft robots. As shown in **Figure** **3**a, the rigid objects, such as fixing rings, screws, nuts, and rigid connector, are employed to connect the two bottom/top facets of artificial muscles, respectively. Screw connector offers notable advantages of reliability, stability, and dismountability. In Figure 3b, soft sucker connector, which has the same driving source as artificial muscles, is used to connect two artificial muscles, enabling the efficient integration of multiple functions in a single soft robot. Soft connection imparts fully soft feature, convenient assembly, and detachment to the combined artificial muscles. Also it can accelerate the testing of new designs, the exploration of new capabilities, and the repair or replacement of damaged parts.

**Figure 3:**
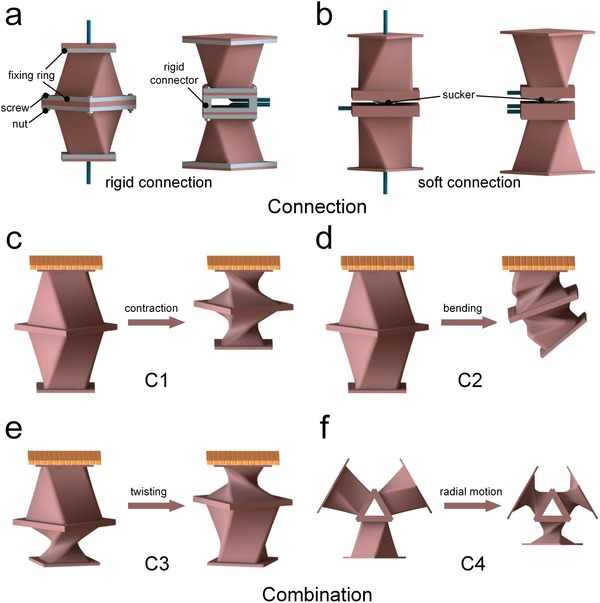
The connection of artificial muscles and motions of combinations. a) Rigid connection. b) Soft connection. c) Contraction motion. d) Bending motion. e) Twisting motion. f) Radial motion.

*Motions of Combined Artificial Muscles*: A single TCAM or TBAM can only perform compound motions: twisting‐contraction or twisting‐bending. Nevertheless, the single motions, such as twisting, contraction, bending, etc., are commonly needed in most applications, instead of compound motions of TCAM and TBAM. Interestingly, the single motions can be decoupled by using different combinations of artificial muscles. As shown in Figure 3c–f, various combinations of artificial muscles, including C1, C2, C3, and C4, can perform contraction, bending, twisting, and radial motions, respectively. Among them, C1 consists of two TCAMs where the top facet of the upper artificial muscle is fixed, and the top facet of the lower artificial muscle is free to move. These two TCAMs twist adversely (anticlockwise and clockwise) and the bottom facets of the two TCAMs are connected together. When two TCAMs are evacuated simultaneously, the length of C1 decreases and the twisting motion is counteracted. Thus, the C1 can perform a single contraction motion with a double displacement. In C2, two TBAMs are combined in the similar way as C1. When the two TBAMs are evacuated simultaneously, the twisting motion is counteracted and a bending motion with a double bending angle is yielded. C3 has a similar configuration to C1 except that the two TCAMs are actuated with different sequence. In C3, only a single TCAM is evacuated at a time, and a twisting motion with a double twisting angle and unchanged length of C3 is realized. For C4, three TCAMs are evenly spaced in a cylindrical configuration and the radial motion is realized by simultaneous actuation of three artificial muscles. All these four combinations can function similarly either by connecting the top or bottom facets together. In addition to these four motions, other simple or complex motions could also be realized by the combined artificial muscles and actuating them with different sequences.

Based on the motions of aforementioned combinations, four soft robots with different functions are designed and reconfigured to complete various tasks, which is as interesting as “building bricks”.

*A Modular Quadruped Robot*: A modular quadruped robot with omnidirectional motion is fabricated by combining multiple artificial muscles. The quadruped robot is square and composed of four scalable modules, four feet, and four connection modules where the top facets of all TCAMs are connected (**Figure** **4**a). A clockwise and an anticlockwise TCAMs form a scalable module, in which two bottom facets are connected using screw connection. When evacuated, the length of the scalable module decreases without relative rotation between the two top facets (see C1 in Figure 3c). Foot is a special TCAM with a sucker mounted at its bottom facet (Figure S7, Supporting Information). As shown in Video S3 (Supporting Information), the quadruped robot can achieve omnidirectional movement using crawling gaits and turning movement using rotating gaits. The average moving speed is 10.7 mm s^−1^, which is equivalent to 3.4 times of its body length per minute. The average turning speed is 18.0° s^−1^, which is faster than most known soft crawling robots, as indicated in Table S2, Supporting Information.

**Figure 4:**
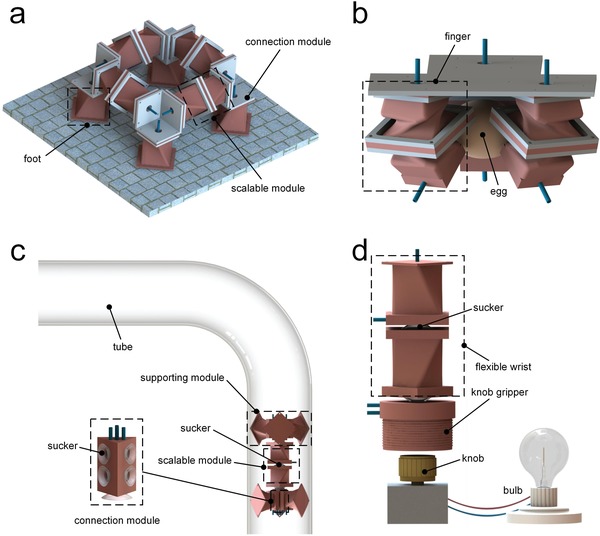
Reconfigurable soft robots. a) The modular quadruped robot. b) The flexible gripper. c) The pipe‐climbing robot. d) The flexible wrist.

*A Flexible Robot Gripper*: A flexible robot gripper is constructed by attaching three fingers to a board made of polylactic acid, as shown in Figure 4b. A clockwise and an anticlockwise TBAMs are combined via screw connection to form a finger. When evacuated, the finger bends to the direction with least infill and no relative rotation is observed between the two top facets (see C2 in Figure 3d), thus the gripper can grasp objects, as shown in Video S4, Supporting Information.

*A Pipe‐Climbing Robot*: An entirely flexible pipe‐climbing robot is made of a scalable module and two supporting modules, as shown in Figure 4c. Each supporting module consists of three TCAMs evenly spaced in a cylindrical configuration (see C4 in Figure 3f), and all artificial muscles are combined together by sucker connectors. The scalable module provides the robot with a driving force by contracting and elongating. The supporting module prevents the robot from sliding in the pipe when unactuated, and decreases the friction between the robot and pipe by reducing its diameter when actuated. Actuating and releasing the scalable module and two supporting modules in a sequence enables the robot to walk along the pipe, as shown in Video S5, Supporting Information. The pipe‐climbing robot is able to climb along a crooked pipe with an inside diameter of 100 mm, at a speed of 7.5 mm s^−1^ (2.14 body length min^−1^).

*A Flexible Robot Wrist*: An entirely flexible robot wrist is fabricated to control the flexible knob gripper, thereby adjusting the brightness of a bulb, as shown in Figure 4d. The wrist is composed of a clockwise and an anticlockwise TCAM, of which the bottom facets are connected together using sucker connector. The flexible knob gripper, which will expand inward and grasp the knob when evacuated, is a fiber‐reinforced circular structure with an inner circular chamber. The actuation sequences of turning up (clockwise rotation) and turning down (anticlockwise rotation) the bulb are shown in Video S6, Supporting Information.

In summary, this work has successfully provided an origami design to impart reconfiguration to flexible material‐based soft robots. Two novel origami artificial muscles (TCAM and TBAM) can perform different compound motions (twisting‐contraction and twisting‐bending) due to their different internal structure. The single motions, including contraction, bending, twisting, and radial motions, are realized through different combinations of artificial muscles. Based on multiple combinations of artificial muscles, four different soft robots, including an omnidirectional quadruped robot, a flexible gripper, a flexible wrist, and a pipe‐climbing robot, are reconfigured, thereby realizing the “building bricks” of soft robots. The robots allow simpler, less expensive, modular units to be reconfigured into a group depending on the task that needs to be completed while being as effective as a larger, task‐specific, monolithic robot. This origami design contributes to the applications of flexible material‐based robots, and has great prospects to be employed in soft robots made by various flexible materials, including hydrogel, electroactive polymer, liquid metal, and so on.

## Conflict of Interest

The authors declare no conflict of interest.

## Supporting information

Supplementary

Supplementary

Supplementary

Supplementary

Supplementary

Supplementary

Supplementary
