# Supplementary material for: Advanced Artificial Muscle for Flexible Material‐Based Reconfigurable Soft Robots
Source: Adv Sci (Weinh). 2019 Sep 4;6(21):1901371. doi: 10.1002/advs.201901371 (PMC6839643; doi:10.1002/advs.201901371)
Supplement: Supplementary file 1 — Supplementary [file ADVS-6-1901371-s002.pdf]

Copyright WILEY-VCH Verlag GmbH & Co. KGaA, 69469 Weinheim, Germany, 2019.

Supporting Information

for *Adv. Sci.,* DOI: 10.1002/advs.201901371

Advanced Artificial Muscle for Flexible Material-Based Reconfigurable Soft Robots

*Zhongdong Jiao, Chao Zhang, Wei Wang, Min Pan, Huayong Yang, and Jun Zou**

WILEY-VCH

Copyright WILEY-VCH Verlag GmbH & Co. KGaA, 69469 Weinheim, Germany, 2019.

ADVANCED SCIENCE
Open Access

Supporting Information

Advanced Artificial Muscle for Flexible Material-based Reconfigurable Soft Robots

Zhongdong Jiao,[†] *Chao Zhang,*[†] *Wei Wang, Min Pan, Huayong Yang, Jun Zou**

Contents:

The fabrication of artificial muscles

Performance test of artificial muscles

Setup for deformation responses test

Pressure control system

The dynamic responses of artificial muscles

The actuation sequences of pipe-climbing robots

The actuation sequences of flexible wrist

The actuation sequences of omnidirectional quadruped robots

Table S1. Details of the soft building bricks robots in this paper.

Table S2. A comparison of the average turning speed of pneumatic soft crawling robots.

Table S3. The shape recovery performance of TCAM.

Table S4. The shape recovery performance of TBAM.

Figure S1. Schematic outlining the fabrication process of artificial muscles.

Figure S2. Schematic of the setup used for deformation responses test.

Figure S3. The pressure control system used to actuate artificial muscles.

Figure S4. The dynamic responses of artificial muscles.

Figure S5. Schematic and gaits of the pipe-climbing robot.

Figure S6. Schematic and actuation sequence of the flexible wrist and knob gripper.

Figure S7. Schematic and gaits of the modular quadruped robot.

Captions for Video S1 to S6.

References

Other supplementary materials for this manuscript include the following:

Video S1 (.mp4 format). The working principle of artificial muscles.

Video S2 (.mp4 format). The resistance to damage of artificial muscles.

Video S3 (.mp4 format). The motion of the modular quadruped robot.

Video S4 (.mp4 format). The motion of the flexible gripper.

Video S5 (.mp4 format). The pipe-climbing robot climbs in a crooked pipe.

Video S6 (.mp4 format). The flexible wrist adjusts the brightness of a bulb.

The fabrication of artificial muscles

All the artificial muscles were fabricated with elastomer casting. The molds (made of polylactic acid) for elastomer casting were manufactured using a 3D printer (Trianglelab, Dforce 300). As shown in Figure S1, two components of E630 silicone rubber (Shenzhen Hong Ye Jie Technology Co., Ltd.) and pigment were mixed, degassed, poured into the molds, and cured at 65 °C for 30 min. Then the artificial muscle body (including slanted sides, curved facets, and top facet) and the bottom facet were removed from the molds after they were cooled to room temperature. Finally, silicone adhesive (Smooth-On, Sil-Poxy) was used to stick silicone tube to the vent hole of the body and stick the body to the bottom facet.

In rigid connection, the fixing rings and rigid connectors were manufactured using a 3D printer. As for the soft connection, the part that sucker is embedded in was made of same material as the artificial muscles. The other part that is sucked by sucker was made of harder silicone rubber (Shenzhen Hong Ye Jie Technology Co., Ltd., E660).

Performance test of artificial muscles

In the deformation performance tests, we fabricated three identical artificial muscle samples for TCAM and TBAM and each sample was tested three times. The height of TCAM and TBAM is 40 mm, the pre-twisted angle of TCAM and TBAM is 45 °, the filling angle of TBAM is 30°. Acrylic sheets were glued to the top and bottom facets to facilitate the measurement. The pressure was regulated using a vacuum regulator (SMC, IRV 1000 regulator) and measured using a pressure sensor (Beijing Star Sensor Technology Co., Ltd., CYYZ31). In the damage tests, a needle with a diameter of 0.71 mm was used to prick artificial muscles.

Setup for deformation responses test

The setup used for the twisting angle and contraction deformation responses of a TCAM is shown in Figure S2a. The deformation process was recorded using two cameras at 60 frames per second. The two cameras were placed perpendicular to the twisting direction and contraction direction, respectively.

The setup used for the twisting angle and bending angle deformation responses of a TBAM is shown in Figure S2b. One camera, which was placed on top of the TBAM, was used to record the twisting process at 60 frames per second. The bending angle was recorded using an inclination sensor (MMA8451), whose data was transmitted to a laptop via the Arduino board (MEGA2560 R3).

Pressure control system

All the modular robots mentioned in the manuscript were controlled using the pressure control system shown in Figure S3. The control signals, generated by the Arduino board (MEGA2560 R3), were transmitted to relays that control the solenoid valves (Kamoer, KVP04 valve) directly. The three-way solenoid valves determine the actuating and releasing of artificial muscles. Vacuum and the atmospheric air (or pressurized air) are connected to the two inlet ports of solenoid valve, respectively, and the artificial muscle is connected to the outlet port. The pressurized air is generated by the air compressor (Jaguar, FB-36/7 compressor) and adjusted using a pressure-regulating valve (Delixi, AFR2000 series). The vacuum is produced by a vacuum pump (VALUE, V-I240SV pump) and regulated using a vacuum regulator (SMC, IRV 1000 regulator). The soft robots are controlled according to the actuation sequences in Figure S5-7.

The dynamic responses of artificial muscles

The dynamic responses of artificial muscles are studied by repeating actuation ten times (the period is 4 s) to observe their shape recovery performance before and after the operation. The

artificial muscles have good shape recovery and deformation performances in these 10 cycles, as shown in Table S3-4.

The shape recovery performance of TCAM:

$$T_{ri} = \frac{|\theta_{nr} - \theta_0|}{\theta_{1d}} \times 100\% , \ C_{ri} = \frac{|\Delta H_{nr} - \Delta H_0|}{\Delta H_{1d}} \times 100\% ,$$

T_{ri} is the twisting recovery index, C_{ri} is the contraction recovery index.

As shown in Figure S4a, θ_{nr} is the twisting angle after cycle *n* (unactuated state), ΔH_{nr} is the contraction after cycle *n* (unactuated state), θ_{1d} is the twisting angle of cycle *1* (actuated state, θ_{1d} = 83.795 deg), ΔH_{1d} is the contraction of cycle *1* (actuated state, ΔH_{1d} = 16.418 mm), θ_0 is the twisting angle before cycle *1* (unactuated state, θ_0 = 0 deg), ΔH_0 is the contraction before cycle *1* (unactuated state, ΔH_0 = 0 mm).

The shape recovery performance of TBAM:

$$T_{ri} = \frac{|\theta_{nr} - \theta_0|}{\theta_{1d}} \times 100\% , \ B_{ri} = \frac{|\beta_{nr} - \beta_0|}{\beta_{1d}} \times 100\%$$

T_{ri} is the twisting recovery index, B_{ri} is the bending recovery index.

As shown in Figure S4b, θ_{nr} is the twisting angle after cycle *n* (unactuated state), β_{nr} is the bending angle after cycle *n* (unactuated state), θ_{1d} is the twisting angle of cycle *1* (actuated state, θ_{1d} = 56.614 deg), β_{1d} is the bending angle of cycle *1* (actuated state, θ_{1d} = 15.800 deg), θ_0 is the twisting angle before cycle *1* (unactuated state, θ_0 = 0 deg), β_0 is the bending angle before cycle *1* (unactuated state, β_0 = 0 deg).

The actuation sequences of pipe-climbing robots

Actuating and releasing the scalable module and two supporting modules in a sequence enables the robot walk along the pipe, as illustrated in Figure S5b-d and Video S5. When the robot is climbing forward, the diameter of the lower supporting module decreases first (S1),

followed by the contraction of the scalable module in S2. The robot moves along the pipe and drives the lower supporting module moving forward. In S3, the lower supporting module is connected to atmospheric pressure and returns to its original shape, effectively attaching to the pipe. Then the upper supporting module is evacuated and its diameter is decreased (S4). The scalable module is connected to atmospheric pressure and returns to its original shape in S5 to drive the upper supporting module moving forward. In S6, the upper supporting module is connected to atmospheric pressure and its diameter increases. Repeating S1 to S6, the robot can continuously climb forward in the pipe. Similarly, the robot can climb backward by repeating S1 to S6 according to Figure S5c-d. The artificial muscles of the pipe-climbing robot weigh 25g, the pipe-climbing robot weighs 440g (17.6 times of the weight of the artificial muscle), the robot can work normally while climbing up a vertical pipe, which indicates that the artificial muscles have good load capacity.

The actuation sequences of flexible wrist

The actuation sequences of turning up (clockwise rotation) and turning down (anticlockwise rotation) the light are shown in Figure S6c-e and Video S6. When the flexible wrist is used to turn up the light, a vacuum of 70 kPa is applied to the TCAM-1 to initialize the twisting action (S0). Then the flexible gripper holds the knob tightly when a high-pressure air of 90 kPa is applied (S1). In S2, we applied -70 kPa to actuate the TCAM-2 and atmospheric pressure to relieve the twisting of the TCAM-1. Both of the two TCAMs offer torque to twist the knob clockwise. In S3 and S4, the gripper loosens the knob and the flexible wrist returns to its initial state. Continuous clockwise rotation by repeating S1-S4 can turn up the light. Similarly, continuous anticlockwise rotation (Figure S6d-e) can turn down the light.

The actuation sequences of omnidirectional quadruped robots

The quadruped robot can achieve omnidirectional movement using crawling gaits (Video S3). As shown in Figure S7c, two adjacent feet are left up (S2, S6) and then dragged (S3) or pushed (S7) forward simultaneously by the contraction or elongation of the scalable modules. Repeating S1 to S8, the robot can continuously move forward. Other crawling gaits, such as moving backward, left, and right are shown in Figure S7d and Figure S7g. The average walking speed is 10.7 mm/s, equivalent to 3.4 times of its body length per minute.

Besides omnidirectional movement, turning movement is also realized in quadruped robot utilizing rotating gaits. As shown in Figure S7e, the two feet that are in a diagonal position are left up (S3, S7) first. Then each raised foot is pushed and dragged by the two adjacent scalable modules simultaneously (S4, S8). Repeating S3 to S10, the robot can continuously turn clockwise. Similarly, the anticlockwise rotating gait can be achieved according to the actuation sequence in Figure S7f and Figure S7h. The average turning speed is 18.0 °/s which is faster than most soft crawling robots[1-5], as shown in Table S2.

Table S1. Details of the soft building bricks robots in this paper.

Applications	Artificial Muscles		Weight (g)
	Numbers	Geometric Parameters	
Quadruped Robot	TCAM (CW[a]) ×6<br>TCAM (ACW[b]) ×6	H_0 = 40 mm	500
Flexible Gripper	TBAM (CW) ×3<br>TBAM (ACW) ×3	H_0 = 40 mm<br>γ = 40°	275
Flexible Wrist	TCAM (CW) ×1<br>TCAM (ACW) ×1	H_0 = 40 mm	206
Pipe-climbing Robot	TCAM (CW) ×4<br>TCAM (ACW) ×4	H_0 = 30 mm	440

[a](clockwise), [b](anticlockwise)

Table S2. A comparison of the average turning speed of pneumatic soft crawling robots.

Soft Crawling Robot	Tethered	Turning Speed (°/s)	Year
Our robot	Yes	18.0	2019
Qin et al.[1]	Yes	15.09	2019
Robertson et al.[2]	Yes	3.5	2017
Waynelovich et al.[3]	No	1.73	2016
Zou et al.[4]	Yes	1.63	2018
Tolley et al.[5]	No	0.20	2014

Table S3. The shape recovery performance of TCAM.

cycle n	θ_{nd} (deg)	θ_{nr} (deg)	T_{ri} (%)	ΔH_{nd} (mm)	ΔH_{nr} (mm)	C_{ri} (%)
cycle 1	83.795	0.545	0.65	16.418	0.005	0.03
cycle 2	83.927	0.652	0.78	16.454	0.001	0.01
cycle 3	84.006	0.754	0.90	16.453	0.020	0.12
cycle 4	83.857	0.797	0.95	16.453	0.018	0.11
cycle 5	83.816	0.807	0.96	16.459	0.029	0.18
cycle 6	84.044	0.812	0.97	16.465	0.026	0.16
cycle 7	83.888	0.875	1.04	16.465	0.025	0.15
cycle 8	83.916	0.812	0.97	16.457	0.026	0.16
cycle 9	83.901	0.825	0.98	16.464	0.030	0.19
cycle 10	83.755	0.879	1.05	16.458	0.025	0.15

Table S4. The shape recovery performance of TBAM.

cycle n	θ_{nd} (deg)	θ_{nr} (deg)	T_{ri} (%)	β_{nd} (deg)	β_{nr} (deg)	B_{ri} (%)
cycle 1	56.614	0.218	0.39	15.800	-0.088	0.56
cycle 2	56.736	0.262	0.46	15.616	-0.087	0.55
cycle 3	56.762	0.274	0.48	15.583	-0.075	0.48
cycle 4	56.760	0.279	0.49	15.557	-0.075	0.47
cycle 5	56.798	0.312	0.55	15.506	-0.081	0.51
cycle 6	56.791	0.316	0.56	15.492	-0.070	0.44
cycle 7	56.886	0.297	0.52	15.492	-0.089	0.56
cycle 8	56.756	0.330	0.58	15.505	-0.073	0.46
cycle 9	56.872	0.308	0.54	15.498	-0.069	0.44
cycle 10	56.902	0.318	0.56	15.442	-0.065	0.41

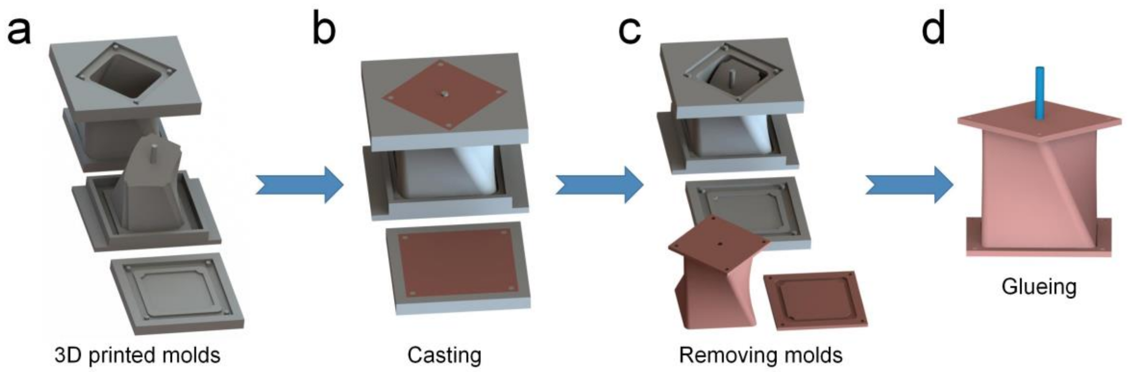

Figure S1. Schematic outlining the fabrication process of artificial muscles. a) The molds utilized to fabricate artificial muscles are printed by 3D printer. b) The silicone rubber is poured into the molds. c) The silicone rubber is removed from the molds after curing at oven. d) The artificial muscle bodies and bottom facets are glued together using silicone adhesive.

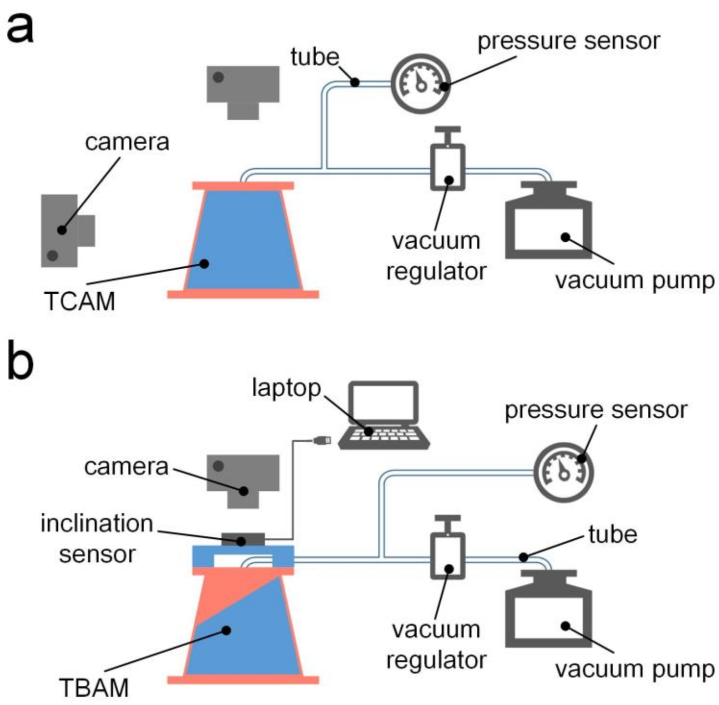

Figure S2. Schematic of the setup used for deformation responses test. a) The deformation response test of TCAM. b) The deformation response test of TBAM.

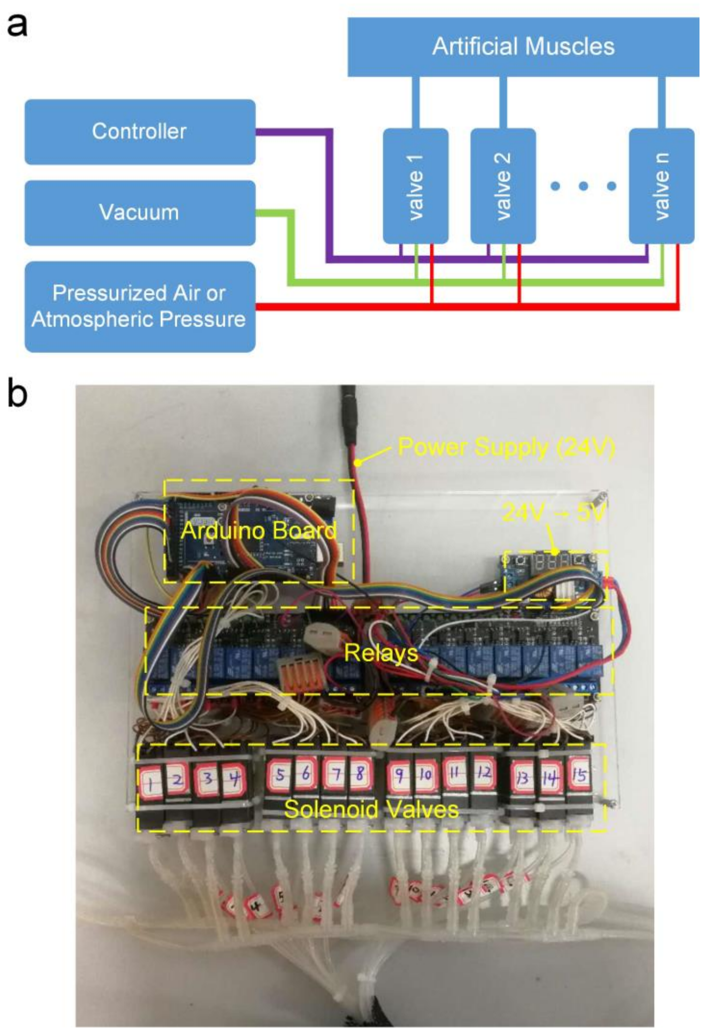

Figure S3. The pressure control system used to actuate artificial muscles.

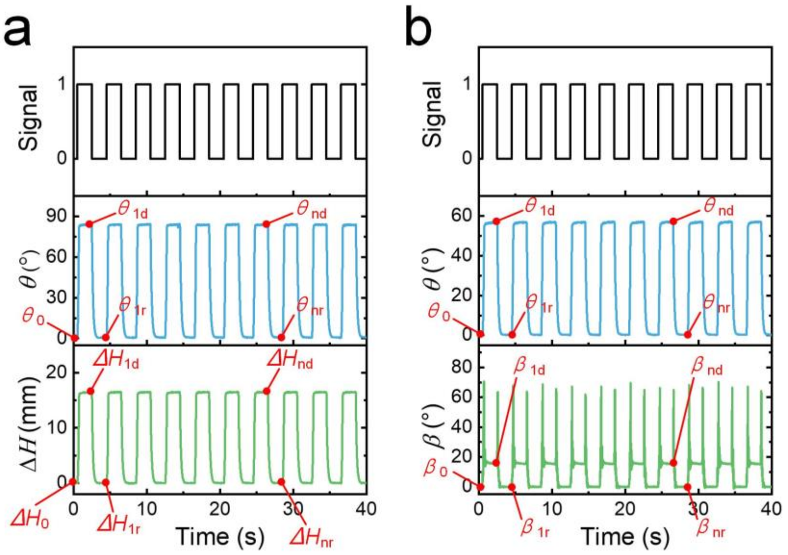

Figure S4. The dynamic responses of artificial muscles.

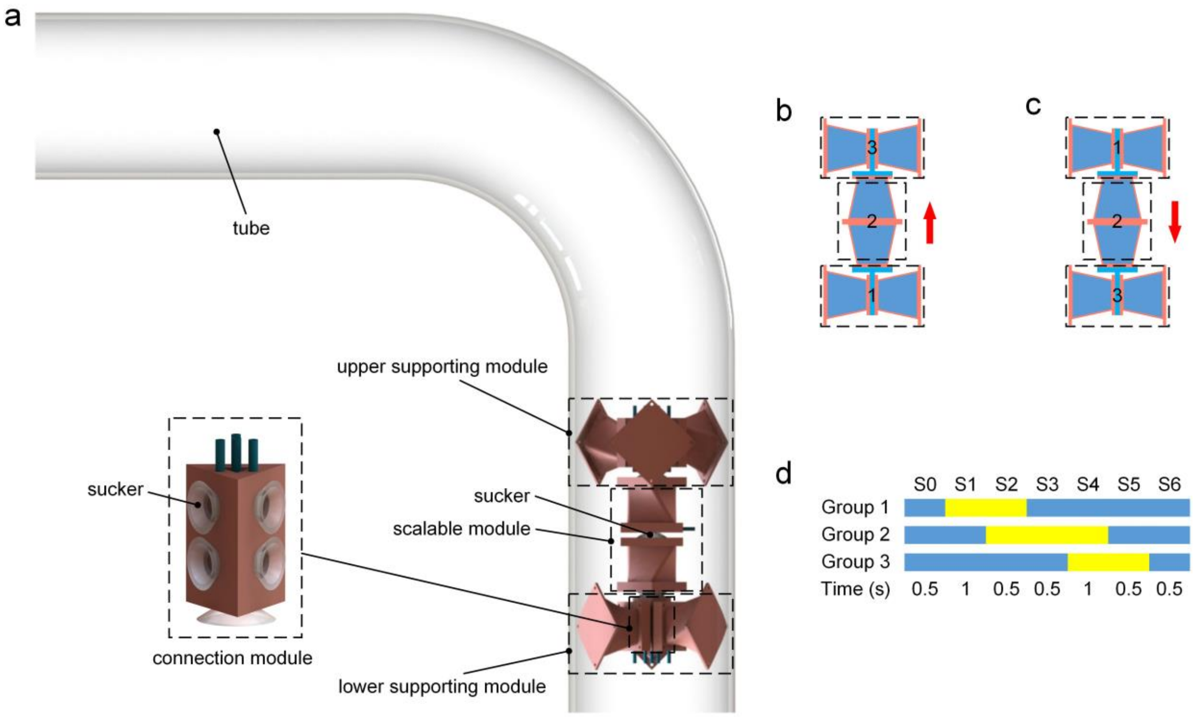

Figure S5. Schematic and gaits of the pipe-climbing robot. a) Schematic of the pipe-climbing robot, which consists of eight TCAMs (four clockwise TCAMs and four anticlockwise TCAMs). Inset depicts a detailed view of the supporting module. b) The grouping diagram of climbing forward in the pipe. Numbers on the model indicate the groups of the artificial muscles. c) The grouping diagram of climbing backward in the pipe. d) Actuation sequence for pipe-climbing robot. The yellow portions indicate actuated TCAMs and the blue portions indicate unactuated TCAMs.

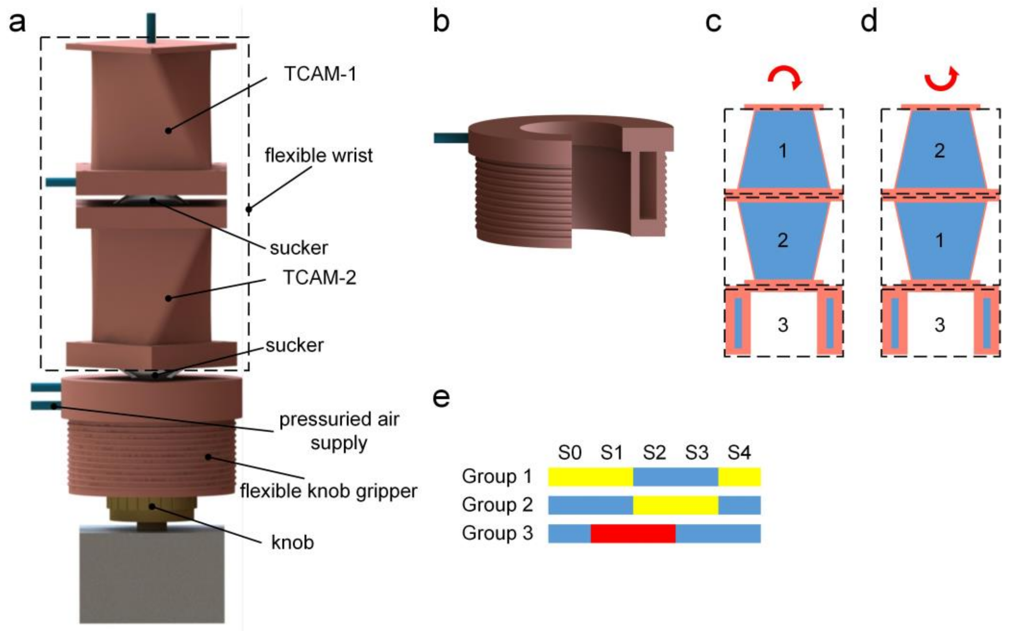

Figure S6. Schematic and actuation sequence of the flexible wrist and knob gripper. a) Schematic of the flexible wrist and flexible knob gripper. Flexible wrist consists of two TCAMs (one clockwise TCAM and one anticlockwise TCAM), whose bottom facets are combined together using sucker connector. b) The inner structure of the fiber-reinforced flexible knob gripper. c) The grouping diagram of turning up the light (clockwise rotation). Numbers on the model indicate the groups of the artificial muscles. d) The grouping diagram of turning down the light (anticlockwise rotation). e) Actuation sequence for flexible wrist and knob gripper. The yellow portions indicate actuated TCAMs, the blue portions indicate unactuated TCAMs or knob gripper, and the red portions indicate actuated knob gripper.

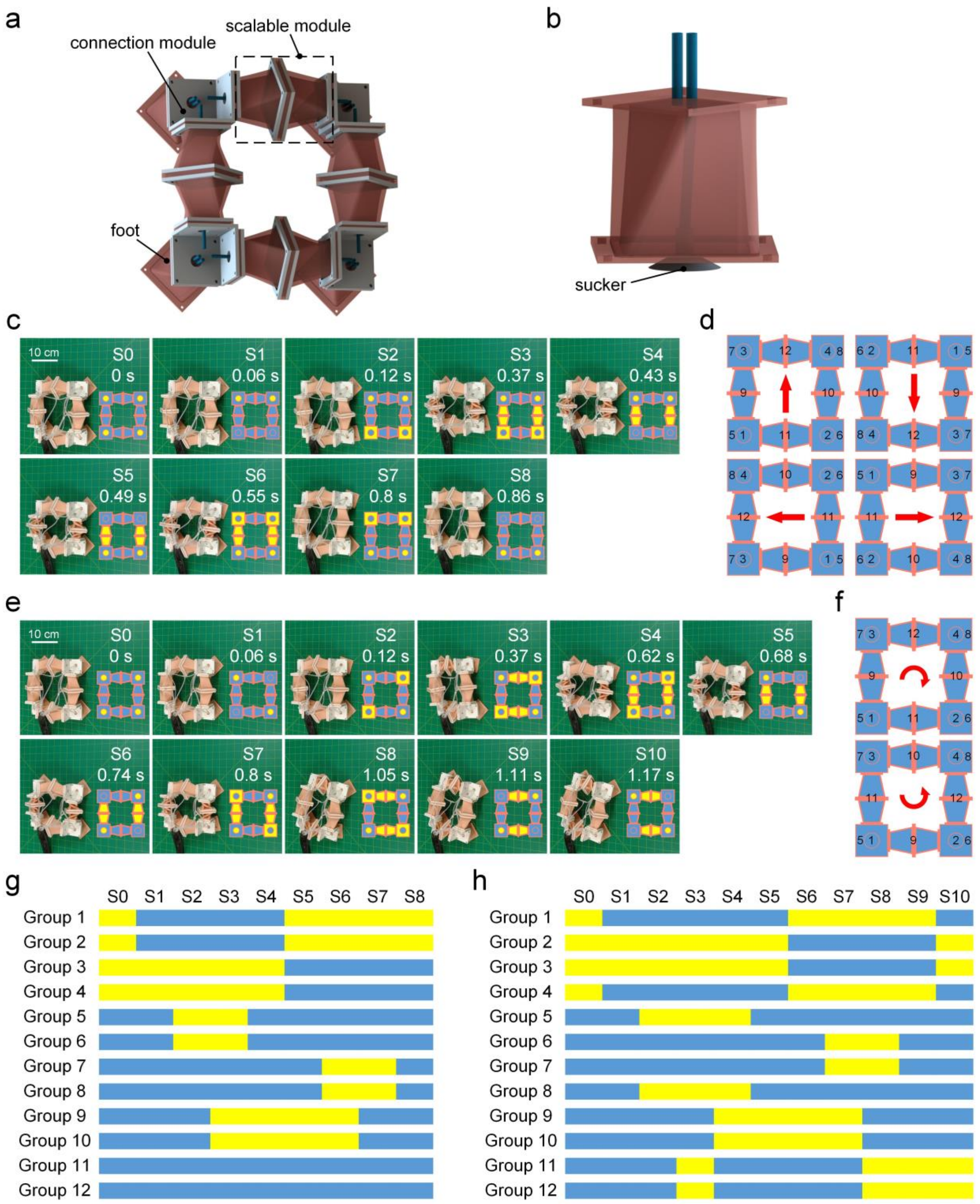

Figure S7. Schematic and gaits of the modular quadruped robot. a) Schematic of the modular quadruped robot, which consists of twelve TCAMs (six clockwise TCAMs and six anticlockwise TCAMs). b) Structure of the foot of the quadruped robot. c) The quadruped robot moves forward using crawling gait. The yellow portions indicate actuated TCAMs or suckers and the blue portions indicate unactuated TCAMs or suckers. d) Four gaits of moving

forward, backward, left, and right. Numbers on the model indicate the groups of the artificial muscles in different gaits. The red arrows indicate the direction of motion. e) The quadruped robot turns clockwise using rotating gait. f) Clockwise and anticlockwise rotating gait. The red arrows indicate the rotation direction. g) Actuation sequence for crawling gaits in (d). h) Actuation sequence for rotating gaits in f).

Video Captions

Video S1. The working principle of artificial muscles. TCAM and TBAM operate with a slow speed to illustrate their working principles and with a normal speed to display their functions.

Video S2. The artificial muscles are hit by a hammer and pricked with a needle (the diameter is 3.6 mm) to demonstrate their resistance to damage.

Video S3. The motion of the modular quadruped robot. The quadruped robot shows its capability of moving forward, moving backward, moving left, moving right, turning clockwise, and turning anticlockwise.

Video S4. The motion of the flexible gripper. The gripper is used to grasp objects.

Video S5. The pipe-climbing robot climbs in a crooked pipe. The pipe-climbing robot is assembled quickly and moves upward, left, right, and downward in a pipe.

Video S6. The flexible wrist adjusts the brightness of a bulb. The flexible wrist is assembled automatically and used to turn up the light first, followed by turning down the light. Then the flexible wrist is disassembled into three parts.

References

[1] L. Qin, X. Liang, H. Huang, C. K. Chui, R.C. Yeow, J. Zhu, *Soft Robot.* **2019**, *6*, 4, 455.

[2] M. A. Robertson, J. Paik, *Sci. Robot.* **2017**, *2*, eaan6357.

[3] J. Waynelovich, T. Frey, A. Baljon, P. Salamon, *Soft Robot.* **2016**, *3*, 64.

[4] J. Zou, Y. Lin, C. Ji, H. Yang, *Soft Robot.* **2018**, *5*, 164.

[5] M. T. Tolley, R. F. Shepherd, B. Mosadegh, K. C. Galloway, M. Wehner, M. Karpelson, R. J. Wood, G. M. Whitesides, *Soft Robot.* **2014**, *1*, 213.
